# A Development-Associated Decrease in Osmotic Potential Contributes to Fruit Ripening Initiation in Strawberry (*Fragaria ananassa*)

**DOI:** 10.3389/fpls.2020.01035

**Published:** 2020-07-10

**Authors:** Kenan Jia, Qing Zhang, Yu Xing, Jiaqi Yan, Luo Liu, Kaili Nie

**Affiliations:** ^1^ College of International Education, Beijing University of Chemical Technology, Beijing, China; ^2^ College of Plant Science and Technology, Beijing University of Agriculture, Beijing, China; ^3^ College of Horticulture, China Agricultural University, Beijing, China

**Keywords:** fruit development, fruit ripening, osmotic potential, water potential, osmotin, strawberry

## Abstract

Fruit development and ripening are accompanied by a large increase in cellular soluble solid contents, which results in a significant decrease in osmotic potential (DOP). Here, we report that this development-associated DOP contributes to the initiation of ripening in strawberry (*Fragaria ananassa* Duch., Benihoppe) fruit. We show that fruit water potential significantly decreases at the onset of ripening as a result of the DOP. Further analysis using nuclear magnetic resonance spectroscopy (NMR) indicated that the change in fruit water potential was likely caused by catabolism of large molecules in receptacle cells, and bioinformatic analysis identified a family of osmotin-like proteins (OLP) that have a potential role in osmolyte accommodation. The gene expression of more than half of the *OLP* members increased substantially at the onset of fruit ripening, and specifically responded to DOP treatment, consistent with a close relationship between DOP and fruit ripening. We report that the DOP induced either by mannitol or water loss, triggered fruit ripening, as indicated by the elevated expression of multiple ripening genes and diverse ripening-associated physiological parameters. Collectively, these results suggest that the DOP contributes to strawberry fruit ripening initiation.

## Introduction

Plant cellular growth involves wall loosening, which results in a reduced water potential that is needed for water uptake and volumetric cell expansion. Although it has been debated whether plant cell growth is primarily controlled by variation in cell wall biomechanical properties or by changes in osmotic pressure (Roy et al., 1998), cellular water status, as determined by hydrodynamics and hydrostatic pressure, is undoubtedly an essential factor controlling cellular growth and differentiation (Proseus et al., 2000; Charras et al., 2005; Mathur, 2006; Proseus and Boyer, 2006; Jia and Davies, 2007). Accordingly, cells have evolved mechanisms to tightly regulate cellular osmolality, membrane tension, and hydrostatic pressure, and changes in cellular hydrodynamics trigger multiple signaling cascades that modify cellular metabolism and physiology (Chaves et al., 2002; Munns, 2002; Chaves et al., 2009).

Changes in cellular water status occur when catabolism results in higher cellular concentrations of metabolites and, consequently, elevated cellular osmotic potential. Large increases in metabolite concentrations rarely occur in plant vegetative organs unless they are exposed to environmental stresses, although there are notable exceptions, such as the ripening of fleshy fruits. The ripening process is complex and involves major changes in a diverse range of biochemical pathways (Bain and Robertson, 1951; Considine and Brown, 1981; Giovannoni, 2001; Seymour et al., 2013; Apri et al., 2014). One such event that is common to many fleshy fruits is the degradation of polymers, such as starch and cell wall polysaccharides, which results in the accumulation of total soluble solids (TSS) (Matthews et al., 1987; Thomas et al., 2006; Luengwilai et al., 2010; Urraca et al., 2016). For example, in ripe grape (*Vitis vinefera)* and strawberry (*Fragaria ananassa*) fruits, soluble sugars accumulate to levels as high as 25% and 15% (800 and 500 mOsm), respectively, of fruit fresh weight (Matthews et al., 1987). Higher TSS levels would be expected to induce a decrease in osmotic potential (DOP), thereby profoundly influencing cellular activities, and influencing physiological and biochemical processes, as well as cell division and differentiation. However, much remains to be learnt regarding changes in fruit water status and the regulation of fruit development and ripening. Of the reports that describe fruit water relations, most are concerned with the effect of the environment on whole plant water status (Kaufman, 1970; Naor et al., 2008), and less is known about how fruit cells accommodate changes in water relationship and how such changes affect cellular biological processes.

Fleshy fruits can be physiologically classified into two major groups: climacteric and nonclimacteric groups (Giovannoni, 2001; Seymour et al., 2013). Climacteric fruits exhibit a burst of respiration and ethylene production, whereas nonclimacteric fruits do not (Giovannoni, 2001; Seymour et al., 2013; Giovannoni et al., 2017), and strawberry provides a model for nonclimacteric fruits (Cherian et al., 2014; Guidarelli and Baraldi, 2015; Haugeneder et al., 2018; Moya-León et al., 2019). While the gaseous hormone ethylene plays a key role in ripening initiation in climacteric fruits, there is increasing evidence that another phytohormone, abscisic acid (ABA), plays an important role in the regulation of strawberry fruit ripening (Moya-León et al., 2019). Molecular studies have identified many structural genes that regulate fruit development and ripening, but much less is known about the mechanisms underlying their regulation. In this regard, key goals are to elucidate the signal transduction pathways that govern fruit development and ripening, as well as the associated primary signals that triggers signaling. Given that fruit development and ripening are accompanied by a DOP, and we hypothesized that this DOP acts as a primary signal for strawberry fruit ripening initiation. This idea is supported by the fact that ABA accumulation is induced by a DOP and that a member of the sucrose nonfermenting 1-related protein kinase 2 (SnRK2) family, which is specifically involved in osmotic signaling (Fujita et al., 2009; Han et al., 2015), plays an important role in the regulation of strawberry fruit ripening.

In this current study, we first analyzed the characteristic changes in fruit water relations related to strawberry fruit development and ripening and showed that a DOP occurs during the ripening phase. To define DOP-associated biological processes in more detail, we characterized the expression patterns of a family of DOP-associated proteins, osmotin-like proteins (OLP), which are known to be tightly associated with the changes in the cellular osmotic potential (Singh et al., 1985; Singh et al., 1987; Singh et al., 1989; Zhu et al., 1993; Sato et al., 1996; Raghothama et al., 1997; Faillace et al., 2019; Pluskota et al., 2019). Through analysis of a range of DOP-associated molecular and physiological events, we provide evidence that the fruit development-associated changes in osmotic potential contribute to the initiation of strawberry fruit ripening.

## Materials and Methods

### Plant Materials and Growth Conditions

Two-month-old strawberry (octoploid: *Fragaria x ananassa*, cv Benihoppe) seedlings, propagated from runners, were transplanted into pots (diameter, 230 mm; depth, 230 mm) containing a mixture of nutrient soil, vermiculite, and organic fertilizer (7:2:1, v/v/v). The seedlings were grown in a controlled environment under the following conditions: 25°C:18°C (day:night), 60% humidity, and a 12-h photoperiod supplied with a photosynthetic photon ﬂux density (PPFD) of 450 µmol m^-2^ s^-1^. The seedlings were well-watered until blooming and fruit set. The first batch of fruit (i.e. the fruits come out at the earliest), corresponding to five specific developmental stages, namely small green (SG), middle green (MG), large green (LG), white (W), full reddening (FR), were used for the experiments.

### Measurement of Fruit Water Status

We adopted a pressure chamber (3000F01 Plant Water Status Console, Soilmoisture Equipment Corp., Goleta, CA, USA) to measure fruit water potential. Fruit at a specific developmental stage were detached from the plant at the stalk base using a sharp razor blade, and the receptacle was immediately sealed in the pressure chamber with a section of stalk protruding from it. Pressure was slowly applied and the water potential value was recorded immediately when exudate appeared at the cut surface. The water potential values in five fruit were measured for each developmental stage. Following each measurement, the fruit was immediately frozen in liquid nitrogen and stored at −20°C until further use for osmotic potential measurements.

Fruit osmotic potential was measured in the same fruit used to measure water potential to more accurately calculate pressure potential using individual fruit. Frozen fruit were thawed at 25°C and cell juice was obtained by centrifuging 7-g samples at 25°C, 2,000*g* for 10 min. Osmotic potential was measured at 25°C using a vapor pressure osmometer (US VAPRO Wescor 5520) calibrated using NaCl solutions, as previously described (Lang, 1967). Osmotic potential was calculated according to the equation: Ψs = –iC*R*T, where Ψs is the osmotic potential, iC is the osmotic concentration (osmolarity), R is the universal gas constant, and T is the absolute temperature. Five fruit were analyzed for each developmental stage. Fruit pressure potential was calculated by subtracting osmotic potential from water potential. Each measurement was analyzed with three biological replicates.

### DWP (i.e., Decrease of Water Potential), DOP (i.e., Decrease of Osmotic Potential) and Phytohormone Treatments

To observe the effect of DWP on gene expression, detached fruit at the MG, LG, and W stages were allowed to lose water in ambient air (temperature 25°C, approximately 30% humidity) for 12 h, and when the fruit weight was ~85% of the original fresh weight, the fruit were sampled for gene expressional analysis by quantitative real time (qRT)-PCR. To observe the effect of DWP on fruit ripening, detached fruit at the MG, LG, and W stagse were allowed to lose water in ambient air (temperature 25°C, approximately 30% humidity) and fruit water potential was monitored at regular time intervals. Once fruit water potential had decreased to −1.8~2.0 MPa, which is the approximate water potential of ripe fruit, the fruit were maintained at 25°C and under 100% humidity to prevent a further decrease in water potential (DWP). Control fruit were maintained under 100% humidity immediately after detachment and the progression of fruit ripening was assessed and the fruit photographed for three biological replicates.

To investigate whether expression of the ripening-associated genes might be able to respond to DOP, fruits at the LG stage were treated by 0.65 M mannitol and the fruit juice derived from fully ripe fruits to mimic the DOP naturally occurred from the LG to the FR stage. To collect the juice from FR fruits, the fruits were first cut into slices of 2 mm in thickness, then frozen in liquid nitrogen and thawed for three times, the juice as a result from the frozen/thawed treatment was collected by centrifugation at 2000*×g* for 10 min. For the DOP treatment, fruit at the LG stage was divided longitudinally into three equal parts and sectioned into slice tissues. The slice tissues were infiltrated by vacuum and incubated fruit juice, 0.65 M mannitol, or distilled water (control) for 5 h in room temperature. For phytohormone treatments, individual receptacle was divided longitudinally into four equal parts and the parts come from three fruits were combined into an individual sample, thus making four samples. The four samples were treated with 100 µM ABA, 100 µM Me-JA, or 0.65 M mannitol, respectively, with distilled water treatment as the control with three replicates. Treated tissues were pooled to minimize variation caused by the different physiological states of individual fruit. Samples were vacuum-infiltrated with treatment solutions for 30 min, and then incubated at 25°C for 5 h. Treated tissue was frozen in liquid nitrogen and subjected to gene expression analysis.

### Measurement of Fruit Firmness

To measure the firmness of the strawberry fruit flesh, a GY-4 fruit hardness tester (Zhejiang TOP Instrument) with a 3-mm probe was used. For each fruit developmental stage, five fruit were combined to form one sample.

### NMR Relaxation Measurements

Water status was determined by observing water mobility using a 2 MHz NMR Rock Core Analyzer (Magritek). The achenes and receptacles of ten fruit were combined to form a single sample for each fruit developmental stage, and for each assay three replicates were analyzed. The NMR moisture content was measured immediately after tissue sampling. The specific experimental parameters were: inter-exp dalay, 10,000 ms; echotime, 500 μh; number of echoes, 1,000; and number of scans, 8. For diploid SG, MG, and W fruits, the specific experimental parameters were: inter-exp dalay: 10,000 ms, echotime: 500 μh, number of echoes: 10,000, and number of scans: 64. For achenes, specific experimental parameters were: inter-exp dalay: 10,000, echo time (:5):500, number of echoes:1,000 μh, and number of scans: 64.

### qRT-PCR Analysis

Primers were designed using Primer3 Plus (http://www.primer3plus.com/cgi-bin/dev/primer3plus.cgi). All primer sequences are listed in **Supplementary Tables S1** and **S2**. Total RNA was extracted from fruit receptacles using an E.Z.N.A.^®^ Total RNA Kit (OMEGA) according to the manufacturer’s instructions. First-strand cDNA was synthesized using 1 μl of total RNA, M-MLV reverse transcriptase (Promega) and oligo(dT) primers in a total volume of 20 μl. qRT-PCR was performed using SYBR Premix Ex TaqTM (TaKaRa) according to the manufacturer’s instructions and a 7,500 Real-Time PCR System (Applied Biosystems). Gene expression levels are presented in the form 2^−△△CT^, where △CT represents the difference between the cycle threshold values of the target and reference genes as described by Jia et al. (2017).

### Determination of Ripening-Related Physiological Parameters

Fruit aroma determination by headspace microsolid phase and gas chromatography-mass spectrometry (GC-MS) was performed by the Beijing Academy of Agriculture and Vegetables Institute. Glucose content was measured using the Shanghai Rongsheng BioPharma Glucose Assay Kit. The levels of sucrose, fructose, citric acid and malic acid weree measured using the Nanjing Institute of Bioengineering kit as described by Jia et al. (2017). Fruit pigment, anthocyanin, total phenol, and flavonoid contents were determined as previously described (Lees and Francis, 1971).

### TSS Measurement

TSS was determined with a handheld sugar refractometer (WYT 0%–80%, Zhangzhou Green-Lake Import & Export Co., Ltd.Fujian, China). For TSS measurement in the fruits from the W to FR stage, saps were obtained by gently squeezing fruit, and for the measurement in the fruits from the SG to the LG stage, fruit was cut into slices and the slices were wrapped and squeezed several times until saps dripped out.

### Statistics

For physiological parameters, each measurement of the parameter consisted of 3–4 biological replicates. For gene expression, each measurement consisted of three biological replicates with three technical replicates. The significant differences were performed with multiple comparisons or the comparison between treatment and control based on the analysis of Student’s t-test (P < 0.05).

## Results

### Changes in Cellular Osmotic Potential Related to Strawberry Fruit Development and Ripening

**Figure 1:**
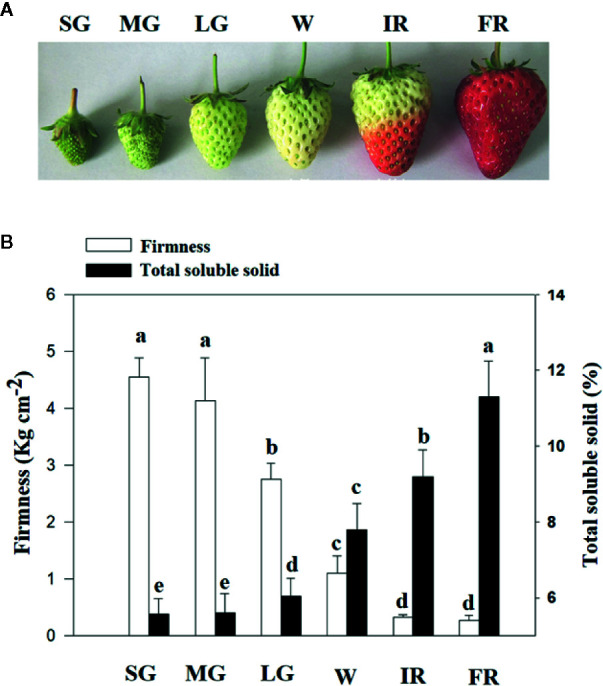
Changes of total soluble solids (TSS) along with fruit development and ripening. **(A)** Phenotypes of fruits at different developmental stages, from fruit set to ripening. Six major developmental stages can be defined: small green (SG), middle green (MG), large green (LG), white (W), and Initial reddening (IR) and Full reddening (FR). **(B)** Changes of TSS (Total Soluble Solid) along with fruit development and ripening. Changes in the fruit firmness indicates the progression of fruit development and ripening. Labels below the bars indicate the corresponding developmental stages as explained in **(A)**. Values are means ± SD of three biological replicates. Different letters denote significant differences at P <0.05 using a Student’s *t*-test.

To characterize the changes in cellular osmotic potential related to strawberry fruit development and ripening, the span of development, from fruit set to ripening, was divided into six major stages: SG, MG, LG, W, and initial reddening (IR) and FR (**Figure 1**). In addition, to quantitatively evaluate fruit development and ripening, fruit firmness was used as a measure of developmental progression, (**Figure 1**). To understand whether changes in TSS might be correlated to the changes in fruit water relationship, we examined the pattern in changes of TSS along with fruit development and ripening. As shown in **Figure 1**, the changes of TSS is tightly correlated with that of fruit firmness, implying a tight correlation with the fruit ripening initiation.

**Figure 2:**
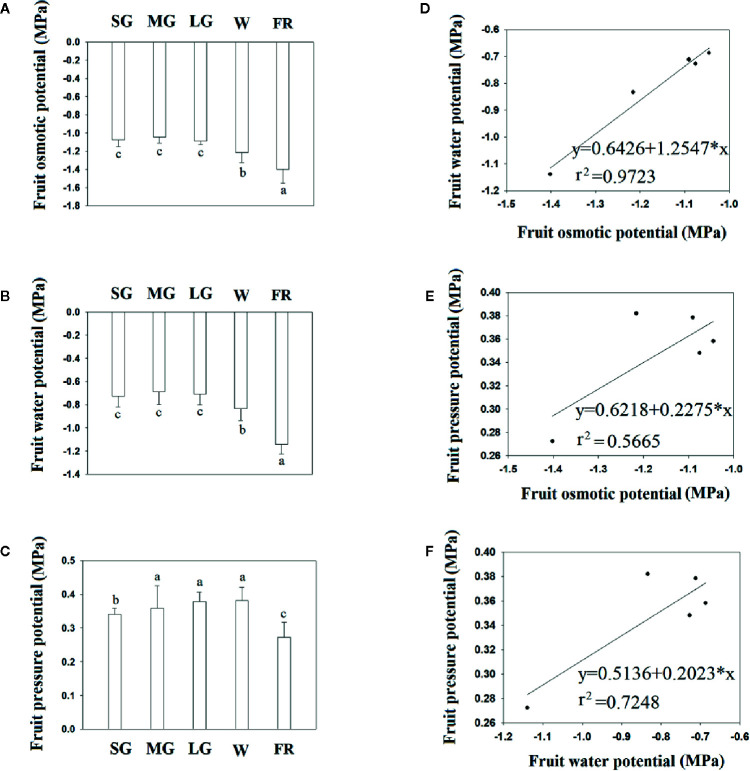
Changes in fruit water status during fruit development and ripening. **(A)** Changes in fruit water potential. **(B)** Changes in fruit osmotic potential. **(C)** Changes in fruit pressure potential. **(D–F)** Correlation analysis of the different water relationship parameters. **(D)** Osmotic potential vs. water potential. **(E)** Osmotic potential vs. pressure potential. **(F)**. Pressure potential vs. osmotic potential. SG, small green; MG, middle green; LG, large green; W, white; and FR, full reddening. In **(A–C)**, bars denote the mean ± SD of four biological replicates. Different letters denote significant differences as determined by a Student’s t-test, P ≤ 0.05.

**Figure 3:**
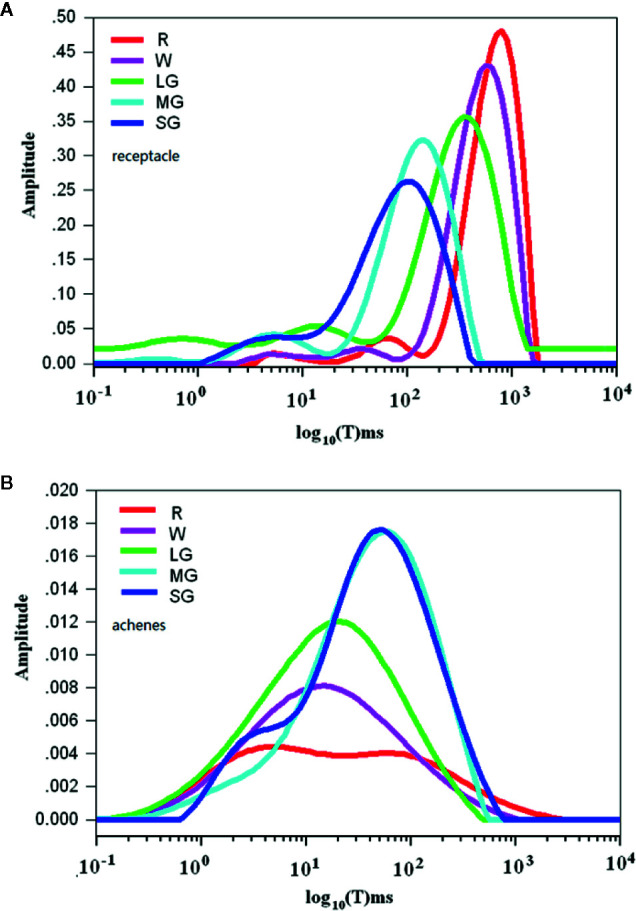
Changes in water mobility during fruit development and ripening as detected by nuclear magnetic resonance (NMR) analysis. **(A)** Spectra showing water mobility status in the receptacles. **(B)** Spectra showing water mobility status in the achenes. The signal amplitude on the Y axis signifies the relative amount of water and the relaxation time on the X axis signifies water mobility. SG, small green; MG, middle green; LG, large green; W, white; and FR, full reddening. Spectral values are the means of hour biological replicates.

**Figure 2** shows the changes in fruit water relation during fruit development. Overall, fruit water potential decreased as fruit developed, although the water potential did not change between the SG to LG stage, and decreased substantially from the W to R stage, indicating that a decrease in fruit water potential is associated with the onset of ripening. **Figure 2** shows the fruit osmotic potential at different developmental stages and, as with water potential, both fruit osmotic potential and pressure potential significantly changed from the LG to W and from the W to R stage. However, unlike the osmotic and water potentials, the pressure potential did not show a significant change from the SG to W stage (**Figure 3**). Correlation analysis showed that the change in osmotic potential was closely associated with the water potential, as evidenced by the high Coefficient of Determination (**Figure 2**, r^2^ = 0.9723). However, we observed no correlation between the osmotic potential and water potential, or between the water potential (**Figure 2**) and pressure potential (**Figure 2**), as indicated by the much lower Coefficients of Determination (r^2^ = 0.5665 and r^2^ = 0.7248, respectively). Collectively, the results above suggest that a decrease in osmotic potential would unavoidably arise from the TSS accumulation during fruit ripening, and that the decrease in osmotic potential would again result in the changes in fruit water relationship.

### Changes in Water Mobility Related to Fruit Development and Ripening

Theoretically, aside from osmotic potential, cellular water relationship can be also affected cellular matric potential, a parameter that is essentially determined by water ability. Contribution of matric potential to water potential is commonly ignored due to its low value (Boyer, 1967; Boyer, 1969). In fruits, the matric potential may possibly affect water potential due to presence of a large amounts of large molecules, such as starch and the cell wall components, to which water molecules might be bound. Accordingly, we analyzed the changes of water mobility during fruit development and ripening using nuclear magnetic resonance spectroscopy (NMR). During fruit development and ripening, fruit water potential decreased in both the receptacle and the achenes, whereas the changes in water content were different between them. **Figure 3** shows rhe pattern of change in the receptacle, where the X-axis shows the signal amplitude signifying the relative amount of water and the Y-axis shows the relaxation time signifying water mobility. The relaxation time greatly increased as fruit development progressed, suggesting that the water status shifted from bound to free in the receptacle. However, in the achenes the relaxation time of the wave peak significantly decreased rather than increased during development and ripening (**Figure 3**).

These observations suggest that the reason for the changes of water potential in the receptacle might be different from that in the achenes. In receptacle, the decrease in cellular water potential is likely caused by the degradation of polymers and hence the increase in TSS, which was consistent with the idea that the degradation of polymers and increase in TSS are important determinants of DOP during fruit ripening. In the achenes, however, the decrease of the cellular water potential is mainly determined by the decrease of water content and the accumulation of macromolecules.

### DOP Promotes Expression of Genes From the OLP Family

**Figure 4:**
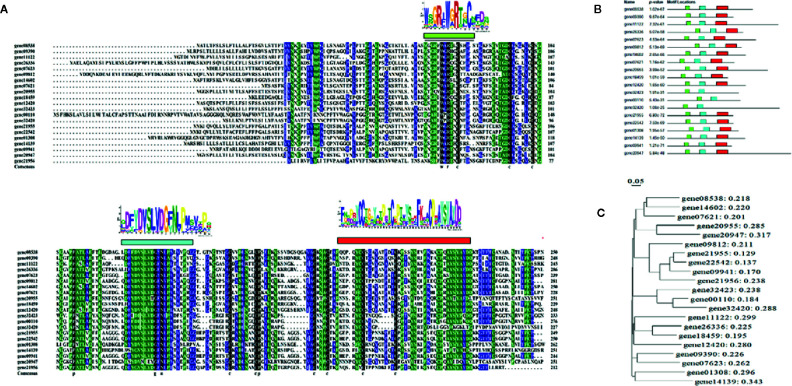
Characterization of the osmotin-like proteins (OLP) family. **(A)** Sequence alignment of the OLP protein family. The deduced amino acid sequences of OLPs were aligned using DNAMAN software (v 6.0) with default settings. Logos above the alignment are the conserved domains searched by the Motif-based sequence analysis tools (The MEME Suite 5.1.1, http://meme-suite.org/index.html). **(B)** motif locations for each OLP member. **(C)** phylogenetic analysis of OLP proteins by DNAMAN software (v6.0).

**Figure 5:**
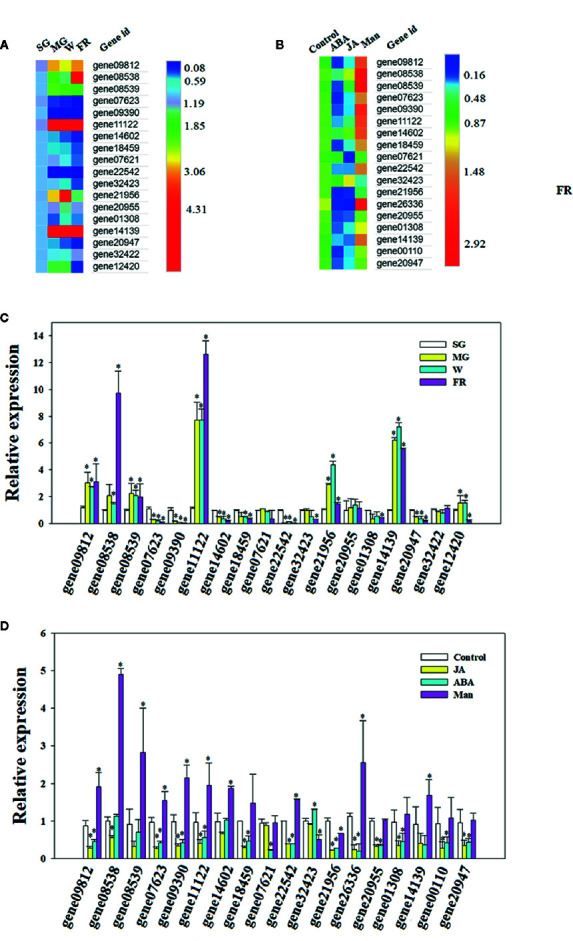
Osmotin-like protein (OLP) expression during fruit development or in response to phytohormones and decrease in osmotic potential (DOP). **(A, B)** Heatmap showing the pattern of gene expression as analyzed by quantitative real time (qRT)-PCR **(C, D)**. Numbers on the right side of the bars denote the fold changes. For abscisic acid (ABA), jasmonic acid (JA) and mannitol treatments (Man), the receptacles at the LG stage were cut into slices and incubated in 100 µM ABA, 100 µM Me-JA or 0.65 M mannitol for 6 h. Controls were SG for the developmental stage incubation in double distilled water (DDW) for the phytohormone and DOP treatments. qRT-PCR values were normalized to the expression value for *FaACTIN* and expressed as fold changes compared to the control. Bars are the mean ± SD of 3 biological replicates. Asterisks denote significant differences compared with the control at *P < 0.05, according to a Student’s *t*-test. SG, small green; LG, large green; MG, middle green; W, white and FR, full reddening.

A search of the strawberry genome identified a family of OLPs, consisting of 23 members (**Table S1** and **Figure 1**). Expression analysis showed that 18 of these were highly expressed in the strawberry receptacle and among these, the expression of 4 showed a substantial increase in expressing during fruit development and ripening (**Figures 5A**). **Figures 4B** show the expression of *OLP* genes**in response to various internal [e.g. the phytohormones, ABA and jasmonic acid (JA)] and external (i.e. osmotic stress) factors that are known to affect strawberry fruit ripening. Treatments with ABA and JA were not found to significantly promote *OLP* expression. Surprisingly, the expression of more than half of the *OLP* genes was substantially higher in response to mannitol treatment (**Figures 5B**), consistent with a relationship with DOP. Collectively, these results suggest that fruit development-associated DOP is likely involved in the transcriptional regulation of many ripening-associated genes.

### DOP Promotes the Expression of Fruit Ripening-Related Genes

**Figure 6:**
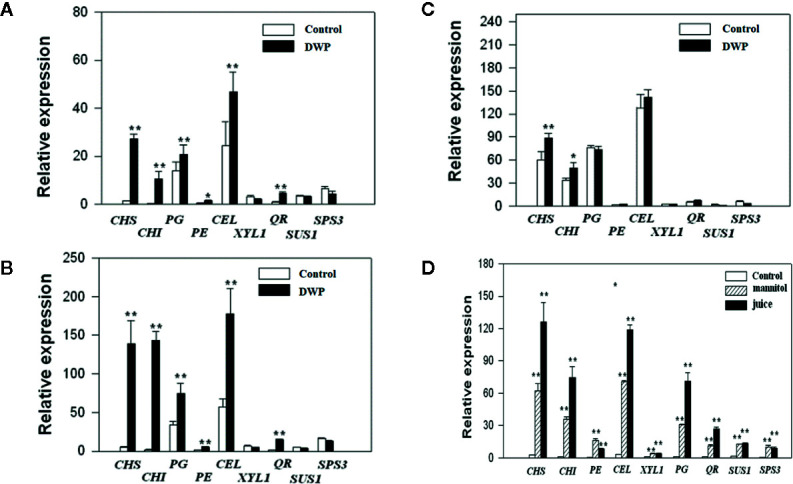
Expression of ripening-related genes in response to decrease in water potential (DWP) or decrease in osmotic potential (DOP) treatments. For DWP treatments, fruit at different developmental stages were allowed to lose water under ambient conditions for 12 h, and when the fruit weight was ~ 85% of the original fresh weight, the fruit were then sampled for gene expressional analysis by quantitative real time (qRT)-PCR. For DOP treatments, receptacles at the large green (LG) stage were cut into slices and incubated for 5 h in 0.65 M mannitol or juice derived from fully ripened fruits (see Materials and Methods for detailed information). Controls were no DWP or DOP treatment. qRT-PCR values were normalized to the expression values of *FaACTIN* and expressed as the fold changes of the control. **(A)** Fruit at the middle green (MG) stage. **(B)** Fruits at the large green (LG) stage. **(C)** Fruits at the reddening (R) stage. **(D)** Fruits treated with mannitol and juice at the LG stage. Bars are the mean ± SD of 3 biological replicates. Asterisks denote significant differences compared with the control at *P < 0.05 and **P < 0.01, according to a Student’s *t*-test.

As DOP is accompanied by a DWP, to investigate whether DOP might induce fruit ripening, we analyzed the effects of DOP and DWP on the expression of the key genes that contribute to aspects of ripening: color (*CHS* and *CHI*), aroma (*QR*), soluble sugar profiles (*SPS* and *SUS*) and cell wall metabolism *(PG, PE, CEL*, and *XYL*). Gene expression was examined at three different developmental stages: MG (**Figure 6**), LG (**Figure 6**) and W (**Figure 6**). DWP was determined by transient fruit dehydration (**Figure 6**), while DOP was determined by mannitol treatment or treatment of juice extracted from ripe fruit. As shown in **Figures 6A** in response to DWP, the expression of *CHS, CHI, PG*, and *CEL* showed a large increase, and compared with the MG stage, fruit at the LG and W stage were more sensitive than the fruitthose at the SG stage, as evidenced by the much higher level of gene expression in the former. **Figure 6** shows gene expression at the LG stage in response to the DOP. Strikingly, expression of all the genes was found to greatly increase in response to DOP, providing further evidence for an effect of DOP on ripening.

### DWP Contributes to the Initiation of Fruit Ripening

**Figure 7:**
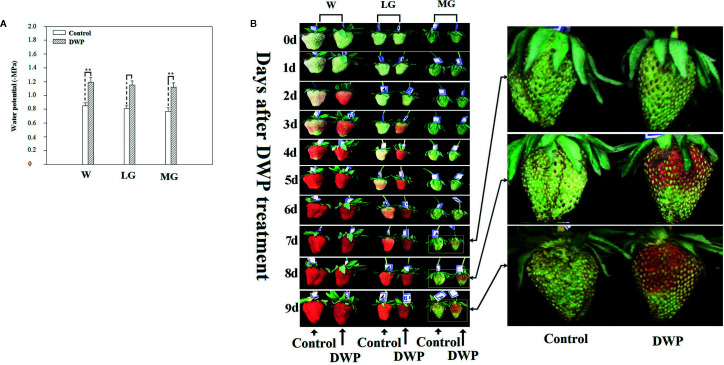
Phenotypic observation of the effect of decrease in water potential (DWP) on fruit ripening. For DWP treatment, detached fruit were allowed to lose water under ambient conditions and fruit water potential was monitored at regular time intervals. Once fruit water potential had decreased to −1.8~2.0 MPa, which is the approximate water potential of ripe fruits, fruit were maintained at 25°C under 100% humidity to prevent further decrease in water potential. Phenotypic changes were observed every day until fruits became red (~10 days). **(A)** Water potential as affected by water loss. **(B)** Phenotypic changes caused by DWP treatment. The right picture shows a magnification of the phenotypic changes in the MG stage. W, white fruit; LG, large green fruit; MG, middle green fruit. Asterisks denote significant differences compared to the control at P < 0.01, according to a Student’s *t*-test.

Although DOP and DWP were shown to promote the expression of fruit-ripening related genes, to demonstrate that the DWP resulting from the DOP contributes to fruit ripening initiation, we analyzed the effect of DWP on several ripening-related physiological and biochemical phenomena. For DWP treatment, detached fruits from the SG, LG, and W stages were allowed to lose water under ambient conditions until the water potential decreased to about 35% of the control within 12 h, and were then incubated in conditions of 100% humidity to stop further loss of water (**Figure 7**). As shown in **Figure 7**, water loss enhanced the rate of fruit ripening, as indicated by changes in fruit color. For example, at the LG stage, desiccated fruit displayed full red coloration between days 5 and 6 of the experimental treatment, whereas control fruit showed only slight red coloration. Fruit water loss resulted in reddening of the fruit at the SG stage, suggesting that DWP affects fruit reddening. In accordance with the reddening progress, other ripening-related physiological parameters were all sensitive to DWP (**Table 1**). DWP treatment resulted in a large increase in sugar content as well as many other parameters. Additionally, DWP treatments also caused a major increase in the content of ABA, a hormone that has been reported to play an important role in the regulation of strawberry fruit ripening (Moya-León et al., 2019; Zhang et al., 2019). Physiological parameters were also highly responsive to both mannitol and fruit juice treatments. As shown in **Table 2**, in response to mannitol or fruit juice treatment, the contents of all sugar components and of flavonoids significantly increased. Moreover, both mannitol and fruit juice treatments caused a large increase in ABA levels. Collectively, these results suggest that DWP, as a result of DOP, contributes to the initiation of strawberry fruit ripening.

**Table 1:** Effects of dehydration treatment on major fruit ripening-related parameters.

Stage	MG		LG		W		Notes
Treatment	Control	Dehydration	Control	Dehydration	Control	Dehydration	
Flavonoid content (μg·g^−1^dry wt)	3.71 ± 0.02	1.65 ± 0.04**	4.01 ± 0.03	1.44 ± 0.01**	1.76 ± 0.01	1.04 ± 0.04**	Pigment metabolism-related compound
Anthocyanin content (μg·g^−1^dry wt)	36.00 ± 3.67	50.01 ± 16.56**	71.38 ± 26.82**	90.03 ± 3.34**	104.13 ± 6.07	109.71 ± 4.08**	
Total phenol content (mg.g^−1^dry wt)	18.33 ± 0.07	24.53 ± 0.01**	20.86 ± 0.07	26.22 ± 0.03**	18.23 ± 0.10	21.90 ± 0.02**	
Sucrose (μmol·mg^−1^dry wt)	55.15 ± 0.51	64.61 ± 2.27**	67.61 ± 0.43	71.01 ± 0.08**	85.75 ± 1.54	88.97 ± 2.29	Sugar and acid metabolism-related parameters
Fructose (mg·ml^−1^dry wt)	69.38 ± 0.95	70.23 ± 1.55	91.48 ± 1.16	93.11 ± 0.50	122.75 ± 0.48	128.78 ± 2.16**	
Glucose (mmol·L^−1^dry wt)	46.51 ± 0.18	47.30 ± 0.05**	46.38 ± 0.31	48.75 ± 0.10**	52.14 ± 0.52	58.80 ± 0.31**	
Citric acid (μmol·g^−1^dry wt)	19.04 ± 0.23	19.13 ± 0.15	21.83 ± 0.10	23.89 ± 0.07**	22.36 ± 0.47	24.17 ± 0.24**	
Malic acid (μmol·g^−1^dry wt)	2.72 ± 0.03	2.73 ± 0.02	3.12 ± 0.01	3.41 ± 0.01**	3.19 ± 0.07	3.45 ± 0.03**	
1-Pentanol	2.33 ± 0.09	2.34 ± 0.01	4.07 ± 0.01	1.87 ± 0.01	1.79 ± 0.01	1.48 ± 0.05**	Aroma metabolism-related compounds (expressed as percentage of total volatiles)
Hexanal	5.84 ± 0.03	6.67 ± 0.04	5.36 ± 0.02	6.42 ± 0.02**	6.83 ± 0.25	4.87 ± 0.12**	Aroma metabolism-related compounds (expressed as percentage of total volatiles)
2-Hexenal, (E1)-	0.22 ± 0.01	0.46 ± 0.02**	0.19 ± 0.01	0.44 ± 0.01**	0.35 ± 0.03	0.24 ± 0.02*	
2-Hexenal, (E2)-	15.21 ± 0.02	30.1 ± 0.02**	15.42 ± 0.01	28.58 ± 0.08**	24.54 ± 0.33	13.91 ± 0.06**	
2-Hexen-1-ol, (E)-	2.94 ± 0.02	1.61 ± 0.04**	2.8 ± 0.05	0.85 ± 0.02**	0.51 ± 0.05	0.85 ± 0.03**	
1-Hexanol	6.11 ± 0.08	8.46 ± 0.04**	8.34 ± 0.01	3.72 ± 0.02**	4.73 ± 0.05	2.18 ± 0.08**	
2,3-Octanedione	0.22 ± 0.01	0.12 ± 0.01**	0.25 ± 0.02	0.12 ± 0.01**	/	0.17 ± 0.01	
1-Octanol	0.35 ± 0.04	0.96 ± 0.02**	0.44 ± 0.02	0.65 ± 0.02**	0.86 ± 0.04	0.77 ± 0.02*	
Nonanal	0.43 ± 0.01	0.34 ± 0.01**	0.4 ± 0.01	0.28 ± 0.02**	0.77 ± 0.02	/	
Decanal	0.12 ± 0.01	0.12 ± 0.01	0.12 ± 0.02	0.1 ± 0.02	0.16 ± 0.01	/	
Acetic acid, hexyl ester	/	/	0.09 ± 0.01	0.33 ± 0.01**	0.68 ± 0.02	0.35 ± 0.02**	
1-Hexanol, 2-ethyl-	/	/	0.13 ± 0.02	0.13 ± 0.02	0.17 ± 0.02	0.16 ± 0.02	

Detached octoploid large green strawberry fruits were used for the analysis. Asterisks denote Student’s t-test significant differences compared with the control: *indicates a significance level of < 0.05; ** indicates a significance level of < 0.01. MG, middle green fruit; MG-de, middle green fruit with dehydration treatment; LG, large green fruit; LG-de, large green fruit with dehydration treatment; W, white fruit; W-de, white fruit with dehydration treatment. Values are means ± SD of three biological replicates.

**Table 2:** Effects of mannitol and fruit juice treatment on major fruit ripening-related parameters.

Parameter	CK	Fruit juice	Mannitol	Notes
Flavonoid content (μg·g^−1^ fresh wt)	0.61 ± 0.01	0.68 ± 0.01	0.64 ± 0.03	Pigment metabolism-related compound
Anthocyanin content (μg·g^−1^ fresh wt)	20.70 ± 0.74	31.50 ± 8.18**	26.10 ± 0.74**	
Total phenol content (μg·g^−1^ fresh wt)	5.07 ± 0.02	6.15 ± 0.08**	5.80 ± 0.06**	
Sucrose (μmol·mg^−1^ fresh wt)	16.95 ± 0.88	20.42 ± 0.45**	22.28 ± 0.09**	Sugar and acid metabolism-related parameters
Fructose (mg·ml^−1^fresh wt)	24.80 ± 1.23	30.46 ± 1.34*	29.38 ± 0.58**	
Glucose (mmol·L^−1^fresh wt)	10.85 ± 0.46	15.23 ± 0.03**	18.22 ± 0.05**	
Citric acid (μmol·g^−1^fresh wt)	4.58 ± 0.36	6.72 ± 0.63**	6.55 ± 0.11**	
Malic acid (μmol·g^−1^fresh wt)	0.65 ± 0.01	0.96 ± 0.09**	0.94 ± 0.02**	

Detached octoploid large green strawberry fruits were used for the analysis. Asterisks denote Student’s t-test significant differences compared with the control: *indicates a significance level of < 0.05; ** indicates a significance level of < 0.01. Values are means ± SD of three biological replicates.

## Discussion

### Arguments About the Measurement of Fruit Water Relationship

In the current study, we performed a comprehensive examination of fruit water relations by measuring multiple parameters, with an emphasis on the changes in fruit water relations associated with ripening. It is challenging to measure fruit pressure potential directly and accurately (Boyer, 1967; Duniway, 1971; Turner et al., 1984). As water potential is mainly determined by osmotic and pressure potential, the most common method to estimate pressure potential (Ψ*p*) is to measure water potential and osmotic potential (Matthews et al., 1987; Wada et al., 2008). Past studies have attempted to directly measure the cell turgor in grape berries using a cell pressure probe (Thomas et al., 2006; Wada et al., 2008); however, successful application of this technique is dependent on the structure of the cell or tissue being measured. The tissue and cell structure of strawberry fruit are different from those of grape, and so we determined the pressure potential using the more commonly used method of measuring water potential and osmotic potential. The order of magnitude of the pressure potential determined in strawberry was approximately 0.3–0.4 MPa, which was comparable to that reported in grape berry (Matthews et al., 1987; Thomas et al., 2006). It was also shown in grape that mesocarp cell pressure decreased at, or near, veraison in *Vitis vinifera* cv. Cabernet Sauvignon and cv. Chardonnay, but not in cv. Cardinal (Matthews et al., 1987). Here, we observed that the pressure potential was static before ripening. Fruit water potential decreased throughout fruit growth and development; however, a major drop occurred after the W stage, suggesting that a DWP was associated more with fruit ripening than with the earlier stages of fruit growth and expansion.

It is known that osmotic potential and pressure potential, as well as the matric potential in certain circumstances, are major factors governing water potential. These factors likely also determine water potential changes during strawberry fruit growth and development. The matric potential is mainly affected by the cell walls (Boyer, 1967) and since cell wall degradation is a critical event in fruit ripening, fruit matric potential may also influence changes in fruit water potential. Matric potential is determined by the bound/free state of water, which is difficult to measure directly; however, NMR can be used to determine water mobility (Biswas et al., 2012 *;* White et al., 2014; Lin et al., 2016). The horizontal axes in **Figure 3** denote relaxation time, which increases when there is more free water. NMR analyses showed that the fruit water status transitioned from the bound to the free state in receptacle tissue during fruit growth and development, but from the free to the bound state in the achenes. It is well established that fruit ripening is accompanied by cell wall degradation, whereas seed development is accompanied by carbohydrate and lipid accumulation. Therefore, the changes in water status seems consistent with the changes that occur in physiological and biochemical metabolism in the receptacle and achenes during fruit development and ripening.

As a transition in water status from the bound to the free state would cause an increase, rather than a decrease, in fruit water potential, the decreased water potential that we observed during fruit ripening did not reflect changes in matric potential, which is determined by water status. Similar, to the changes in water potential, fruit osmotic potential decreased during ripening, especially at the later stages. A correlation analysis indicated that the changes in water potential were strongly associated with those in osmotic potential, with coefficients of determination (r^2^) of 0.9723m. In contrast, a correlation analysis between fruit water potential and pressure potential, and between osmotic potential and pressure potential, revealed coefficients of determination (r^2^) of 0.5665 and 0.7248, respectively. TSS values are known to increase greatly throughout fruit development and ripening. In grape berry, for example, reports showed that the accumulation of solutes, mainly glucose and fructose, may have caused osmotic potentials to reach −3 to −4 MPa (Matthews et al., 1987; Thomas et al., 2006). We concluded that the observed decrease in osmotic potential (DOP) was the result of TSS accumulation. The close correlation between the osmotic potential and water potential indicates that the DWP resulted from DOP. Pressure potential is strongly influenced by the biomechanical properties of the fruit cell walls, which undergo disassembly during ripening, and this supports the observation that there was no correlation between the water potential and pressure potential, or between the pressure potential and osmotic potential.

### Synergistic Action Between the DOP and Phytohormone Signal

As early as to the 1950s, Nitsch demonstrated that the growth of the strawberry receptacle was regulated by the achenes and auxin (Indoacetic acid, IAA) (Nitsch, 1950; Nitsch, 1955). Later work by Given et al. (1988) demonstrated that the transport of IAA from achenes to receptacle plays a critical role in the regulation of strawberry fruit ripening. In recent years, however, it has been increasingly suggested that abscisic acid (ABA) plays an important role in the regulation of strawberry fruit ripening (Chai et al., 2011; Jia et al., 2011; Jia et al., 2013; Kadomura-Ishikawa et al., 2015; Jia et al., 2017; Liao et al., 2018). Intriguingly, besides IAA and ABA, there are evidences that giberellic acid (GA), JA, and ethylene were all implicated in the regulation of strawberry fruit ripening (Csukasi et al., 2011; Symons et al., 2012; Concha et al., 2013; Merchante et al., 2013; Garrido-Bigotes et al., 2018; Gu et al., 2019). As nearly all the phytohormones have been reported to play important roles in the regulation of strawberry fruit development and ripening, it is hard to determine which phytohormone may be the principal signal controlling strawberry fruit ripening. Strawberry fruit ripening is a complex process, which involves dramatic changes in a variety of biochemical metabolisms, such as color, sugar, acid, aroma and cell walls (Lin et al., 2009; Seymour et al., 2013). It is likely that strawberry fruit ripening is regulated by a synergistic action of multiple phytohormones and different combination of the phytohormones may be involved in different process of the biochemical metabolisms. In the current study, we demonstrated that DOP serves as a signal (named as DOP signal). It is well known that ABA biosynthesis can be induced by a decreased water potential. Although it is hard to conclude that DOP is the principle signal controlling strawberry fruit ripening, in the future, it is of significance to demonstrate whether ABA signal might be resulted from the DOP signal.

### Pattern of OLP Expression Supports a Role of DOP in Fruit Ripening

Singh et al. (1987) first identified a protein whose level dramatically increased in response to osmotic stress in cultured tobacco cells, and so named it osmotin. OLP families were later found to be universally present in plants and linked to a diverse range of biological processes, including fruit development and ripening (Singh et al., 1985; Singh et al., 1987; Singh et al., 1989; Zhu et al., 1993; Sato et al., 1996; Raghothama et al., 1997; Faillace et al., 2019; Pluskota et al., 2019). While the OLP family was commonly thought to be related to plant stress responses, a number of studies have shown that the expression of some OLPs respond to phytohormones, such as ethylene, ABA and JA (Singh et al., 1989; Zhu et al., 1993; Raghothama et al., 1997; Pluskota et al., 2019). The responses of *OPL* genes to either osmotic stress or phytohormones suggest roles in metabolic regulation (Faillace et al., 2019). Here, we characterized the expression of members of the strawberry *OLP* family in the context of DOP and in response to treatment with ABA and JA. Although the expression of most *OLP* genes was not induced by either ABA or JA, most showed an increase in response to a DOP, indicating that the *OLP* genes indeed specially responded to DOP There are evidences that the *OLP* genes are tightly associated with fruit ripening (Pressey 1997; Davies and Robinson, 2000; Kim et al., 2002), which implies that DOP should be involved in metabolic regulation during fruit ripening. Collectively, the special pattern of *OLP* expression strongly supports a role of DOP in the regulation of strawberry fruit ripening

### Overall Response of the Ripening-Associated Metabolisms to DOP

As described above, DOP inevitably results in DWP, but DWP can directly be brought about by cellular dehydration and so we observed the effect of DWP on fruit ripening. As expected, DWP strongly accelerated fruit ripening, as indicated by earlier reddening. As shown in **Figure 1**, the transition from fruit set to ripening can be divided into five major stages as indicated by the changes in fruit color. The W (white fruit) stage, also known as the breaking or veraison stage, precedes fruit reddening. The effect of DWP on fruit ripening was sufficiently strong that MG fruit became red without going through the W’ stage, although it is not known whether DWP specifically regulates pigment metabolism, or ripening-related metabolism in general. Further analysis indicated that DWP indeed promoted overall metabolism, as evidenced by the expression promotion of genes associated with color, aroma, sugar and texture (**Figure 5**), as well as a range of physiological and biochemical ripening-related processes (**Table 1**). The decrease in osmotic potential during fruit ripening implies a lower osmotic potential of the juice derived from the ripen fruits than that from the nonripen fruits. To demonstrate whether such as a difference in osmotic potential, alternatively, whether naturally occurring DOP, was enough to promote fruit ripening, we analyzed the responses of fruit at the LG stage to the treatment of the juice derived from the fully reddened fruit. The responses of both gene expression (**Figure 5**) and metabolism (**Table 2**) of the LG fruits to the treatment of the juice derived from the ripen fruits have provided direct evidences fruit development-associated DOP is indeed involved in the regulation of fruit ripening. Accordingly, we propose that the fruit development-associated DOP contributes to the initiation of strawberry ripening.

## Conclusion

TSS increased substantially along with the progression of strawberry fruit development and ripening and this increase in the TSS resulted in a decrease in fruit water potential during fruit ripening. The decrease in fruit osmotic potential acts as a primary signal contributing to the initiation of strawberry fruit ripening.

## Data Availability Statement

The datasets presented in this study can be found in online repositories. The names of the repository/repositories and accession number(s) can be found in the article/**Supplementary Material**.

## Author Contributions

KJ performed most of the experiments. QZ performed some experiments and provided technical assistance. YX contributed to the data analysis. QY, LL, and KN conceived the project. QY drafted the manuscript. LL and KN polished the manuscript. All authors contributed to the article and approved the submitted version.

## Funding

This work was supported by the National Key Research and Development Program (2019YFD1000800) and the Construction of Beijing Science and Technology Innovation and Service Capacity in Top Subjects (CEFF-PXM2019_014207_000032).

## Conflict of Interest

The authors declare that the research was conducted in the absence of any commercial or financial relationships that could be construed as a potential conflict of interest.
