# Supplementary material for: A Development-Associated Decrease in Osmotic Potential Contributes to Fruit Ripening Initiation in Strawberry (Fragaria ananassa)
Source: Front Plant Sci. 2020 Jul 10;11:1035. doi: 10.3389/fpls.2020.01035 (PMC7365926; doi:10.3389/fpls.2020.01035)
Supplement: Supplementary file 1 [file DataSheet_1.docx]

**Supplementary table S1. Characteristics of the conserved domain as analyzed by the CD-search of NCBI**

| **Query** | **Hit type** | **PSSM-ID** | **From** | **To** | **E-Value** | **Bitscore** | **Accession** | **Short name** | **Incomplete** | **Superfamily** |
| --- | --- | --- | --- | --- | --- | --- | --- | --- | --- | --- |
| gene08538 | specific | 334000 | 30 | 247 | 4.3922E-132 | 374.219 | pfam00314 | Thaumatin | - | cl02511 |
| gene09390 | specific | 185757 | 28 | 244 | 3.4079E-124 | 351.543 | cd09218 | TLP-PA | - | cl02511 |
| gene11122 | specific | 185757 | 32 | 241 | 6.9739E-115 | 334.595 | cd09218 | TLP-PA | - | cl02511 |
| gene11122 | specific | 340465 | 328 | 402 | 1.69608E-52 | 169.547 | cd01766 | Ubl_UFM1 | - | cl28922 |
| gene26336 | specific | 334000 | 69 | 283 | 2.1884E-106 | 307.58 | pfam00314 | Thaumatin | - | cl02511 |
| gene26336 | superfamily | 352111 | 27 | 43 | 0.00118493 | 39.4543 | cl04109 | Methyltransf_7 superfamily | NC | - |
| gene07623 | specific | 334000 | 27 | 236 | 2.5943E-118 | 339.936 | pfam00314 | Thaumatin | - | cl02511 |
| gene09812 | specific | 185757 | 61 | 280 | 1.2869E-127 | 361.558 | cd09218 | TLP-PA | - | cl02511 |
| gene14602 | specific | 334000 | 31 | 247 | 5.3632E-124 | 352.648 | pfam00314 | Thaumatin | - | cl02511 |
| gene07621 | specific | 334000 | 10 | 225 | 3.5265E-126 | 356.5 | pfam00314 | Thaumatin | - | cl02511 |
| gene20955 | specific | 334000 | 30 | 253 | 1.1776E-101 | 299.491 | pfam00314 | Thaumatin | - | cl02511 |
| gene20955 | superfamily | 354810 | 315 | 380 | 4.67598E-21 | 91.5654 | cl21453 | PKc_like superfamily | NC | - |
| gene18459 | specific | 334000 | 14 | 227 | 1.8193E-115 | 328.381 | pfam00314 | Thaumatin | - | cl02511 |
| gene12420 | specific | 185757 | 29 | 244 | 7.0168E-116 | 332.283 | cd09218 | TLP-PA | - | cl02511 |
| gene32423 | specific | 128501 | 32 | 230 | 4.2085E-114 | 326.72 | smart00205 | THN | - | cl02511 |
| gene00110 | superfamily | 351780 | 78 | 276 | 1.7361E-105 | 305.149 | cl02511 | GH64-TLP-SF superfamily | - | - |
| gene32420 | superfamily | 351780 | 26 | 189 | 4.44083E-90 | 271.637 | cl02511 | GH64-TLP-SF superfamily | - | - |
| gene32420 | superfamily | 215538 | 189 | 393 | 7.22073E-90 | 280.439 | cl33620 | PLN02996 superfamily | N | - |
| gene21955 | specific | 185757 | 26 | 248 | 1.1857E-109 | 314.564 | cd09218 | TLP-PA | - | cl02511 |
| gene22542 | specific | 185757 | 28 | 249 | 2.1592E-107 | 308.786 | cd09218 | TLP-PA | - | cl02511 |
| gene01308 | specific | 185757 | 40 | 258 | 2.1549E-114 | 327.276 | cd09218 | TLP-PA | - | cl02511 |
| gene14139 | specific | 185757 | 29 | 241 | 3.1136E-106 | 307.631 | cd09218 | TLP-PA | - | cl02511 |
| gene09941 | specific | 185757 | 34 | 243 | 1.8452E-99 | 288.371 | cd09218 | TLP-PA | - | cl02511 |
| gene20947 | specific | 334000 | 30 | 244 | 2.61101E-93 | 282.542 | pfam00314 | Thaumatin | - | cl02511 |
| gene20947 | superfamily | 354810 | 345 | 512 | 1.63245E-59 | 197.495 | cl21453 | PKc_like superfamily | C | - |
| gene21956 | superfamily | 351780 | 21 | 207 | 2.84291E-77 | 230.976 | cl02511 | GH64-TLP-SF superfamily | - | - |

**Supplemental Table S2：Quantitative PCR primers used to analyze the expression of ripening-related marker genes**

| **Gene** | **Forward primers (5’-3’)** | **Reverse primers (5’-3’)** | | **Genbank accession numbers** | |
| --- | --- | --- | --- | --- | --- |
| *FaCHS* | CATACCCCGACTACTACTTTCGT | | CGCACATACTGGGATTCTCTT | | AY997297.1 |
| *FaCHI* | AGCGAAAGCCATTGAAAAGT | | CATTTGGTGATTGTGTGAAGAG | | AB437286.1 |
| *FaPE* | GGTTTCTACTGGTGCTGGTTTT | | CTCGGACTGTATCGTGTTGC | | AY324809.1 |
| *FaPG* | GCAAGTAGAGTCGCACAGTTTT | | TCAGTATTAGGCTTCCCACCA | | DQ45899.1 |
| *FaCEL* | GCTCTGTTTTGCCTGGACTT | | GCGTGGCTTAGATAGTTGGAAT | | AF051346.1 |
| *FaXYL1* | ATGGAAAGCCTACTTGTGCTG | | CTGGTGTAATGTTGTTGGTCGT | | AY486104.2 |
| *FaQR* | CACTGACTCTCCCCTACCTACAAT | | ATACACTTCATCCCCCACCTTA | | AY048861.1 |
| *FaSPS1*  *FaSS1*  *FaACTIN* | CGTAGATTGGAGTTATGGAGAGC  CCCTGATTCTGACCTTTACTGG  GCCAACCGTGAGAAGATG | | CGAATGATGTAAGAACCACTGC  GATGATGAAGTCGGTGTGGTT  TCCAGAGTCAAGAACAATACCAG | | XM_004292927  XM_004303343  XM_011472557.1 |

**Supplemental Table S3：Quantitative PCR primers used to analyze the expression of OLP genes**

| **Gene** | | **Forward primers (5’-3’)** | | **Reverse primers (5’-3’)** | |  |  |
| --- | --- | --- | --- | --- | --- | --- | --- |
|  | |  | |  | | |  |
| \| **gene08538** \| \| --- \| \| gene09390 \| \| gene07623 \| \| gene09812 \| \| gene08539 \| \| gene11122 \| \| gene14602 \| \| gene07621 \| \| gene20955 \| \| gene18459 \| \| gene32423 \| \| gene00110 \| \| gene22542 \| \| gene01308 \| \| gene 26336 \| \| gene14139 \| \| gene20947 \| \| gene21956 \| | \| GCTTCGCAAGGCCAAACCAC  GCCTAGCCTCACTCTCCTC \| \| --- \| \| TATACGGTGACGTTTTGCCCTT \| \| ACGACCAAAACAAGGATGAAGCC \| \| CCGCCGCCAACAGAAACA \| \| CACTCTGACCTCCGATCCA \| \| TAGTCGCTCTCGGTCTCGTTC \| \| AGTCGGCATCGTTCAAGA \| \| TACAGCGTTGTTTTCTGCCCTA \| \| GAGGATGGGGCTCAACTCAT \| \| TGTACCCGAACAGGTTCCTT \| \| CTTCCACAAAAGCCTCGCAGT \| \| GAGCGTGATCGGCTGCATGAG \| \| TGCAGGCCATATGGGAAA \| \| CACAGGCAATGATTTCAATACCCT \| \| CACAGGCAATGATTTCAATACCCT \| \| ACCAGCAGAAACACCATCC \| \| ATGCTGATAATCCGAGTGTTCC \| | | \| GCCAAACAGCCACCGTGAC  GGAGAGTGAAGCCGCCTTTG \| \| --- \| \| CCAGAAGAGCCTCCTGACCC \| \| AATCGAGCTCCTCCGAATAAGGT \| \| AGATTGATGAAGCAGCCATT \| \| GGAATACATTGCCTGCACT \| \| CCATAAACGACCCGACCAAGC \| \| CCGACCATGAATTGGGTACTGAT \| \| CTCTTCCTTCTACTGCTCCAT \| \| AAGGAGCAACCCTGCCTG \| \| TCGACCATCGTCCAACTCC \| \| CTGGTTAAGCTGTTTGCCTCC \| \| CCTTTCTTGTCATCATAAGCG \| \| TGCAGGCCATATGGGAAA \| \| GCAGTTGTTCACTATGATGAGTTG \| \| GCAGTTGTTCACTATGATGAGTTG \| \| TCGACCATCCTCCAACTCC \| \| TTGTACGGCCCCAGAAGTGA \| | |  |  |  |
